# Multi-hazard risk characterization and collaborative control oriented to space in non-coal underground mines

**DOI:** 10.1038/s41598-022-20437-8

**Published:** 2022-09-30

**Authors:** Menglong Wu, Nanyan Hu, Yicheng Ye, Qihu Wang, Xianhua Wang

**Affiliations:** 1 School of Resource and Environmental Engineering, Wuhan University of Science and Technology, 430081, Wuhan, Hubei People’s Republic of China; 2 Sinosteel Wuhan Safety and Environmental Protection Research Institute Co., Ltd, Wuhan, 430081 Hubei People’s Republic of China

**Keywords:** Engineering, Civil engineering, Environmental impact, Hydrogeology

## Abstract

In order to realize accurate risk assessment and collaborative control of multi-hazard risk in non-coal underground mines, a space-oriented risk characterization and collaborative control model of multi-hazard risk in non-coal underground mines is proposed. Statistical analysis of non-coal underground mine accidents from 2000 to 2022, revealing the characteristics of non-coal underground mine accidents and 5 risk types were identified, including cage fall accident, powered haulage accident, fire accident, mine water inrush accident, and roof fall and rib spalling accident. A multi-hazard risk analysis and assessment framework for non-coal underground mines based on the inherent risk of the system, the vulnerability of the disaster-bearing body and the adaptability of the disaster-bearing area is proposed. The multi-hazard inherent risks in non-coal underground mines are comprehensively identified and evaluated in five aspects, including hazardous equipment and facilities, hazardous materials, hazardous processes, hazardous operations and hazardous places, and the characterization and unified measurement of multi-hazard risk is realized by combining the vulnerability index of disaster-bearing body and the adaptability index of disaster-bearing area. Regional multi-hazard risk aggregation is achieved through the Nemerow pollution index and space-oriented multi-hazard risk is obtained. Constructed a multi-hazard safety risk collaborative control system of source identification, classification and control, process control, continuous improvement, and full participation. Finally, the validity and rationality of the risk characterization model and the risk collaborative control system are verified. The research can both support the formulation of macro policies for non-coal underground mines and provide guidance for the specific spatial layout.

## Introduction

The underground mine production system is an extremely complex spatially distributed disaster system. Due to many factors such as high ground pressure, high ground temperature, high shaft depth, high karst water pressure, complex empty areas and fracture zones, underground mining is in an extremely complex and harsh environment. Multi-hazard underground mine as a special mine with greater risk in the mining industry, due to the coexistence of multiple hazards, under the mining disturbance conditions multi-hazard disasters directly or indirectly trigger the occurrence of other hazards in the mining process, so that the production process of multi-hazard underground mines face more serious safety problems. Therefore, the construction of space-oriented multi-hazard source safety risk assessment model and multi-hazard source safety cooperative control system for non-coal underground mines is one of the key research topics for mine safety workers at present.

Risk analysis for multi-hazard sources has received significant attention in recent years^1,2^. However, the concept of multi-hazard risk analysis is quite extensive, and the existing research is different in a wide range of issues. Kappes et al.^3^ defined multi-source risk as “the totality of relevant hazards in a defined area” and divided the analysis methods of multi-source risk into two categories. (i) Spatially oriented and aims at including all relevant hazards; (ii) Thematically defined. Gill et al.^4^ defined multi hazard risk as "a specific situation in which major hazard sources interact with each other over time and may occur simultaneously, cascade or cumulatively". At the same time, the relationship between multi-hazard disasters is divided into two categories: Compound hazards and concurrent or consecutive hazards. Analyzing previous studies related to multi-hazard source risk, in some cases, multi-hazard source risk studies involve hazards that are not dependent or unrelated, but are likely to occur at different spatial distribution points^5–7^; in other cases, research on multi-hazard hazards has focused on hazards that occur simultaneously or are closely related to each other^8–10^.

Looking at the relevant studies on risk identification, index analysis, and risk assessment models at home and abroad, many scholars have conducted numerous studies based on different perspectives and using different analysis methods. Ruan et al.^11^ conducted risk assessment of mine water inrush disaster based on analytic hierarchy process (AHP) and Dempster-Shafer (D-S) evidence theory; Li et al.^12^ achieved a quantitative characterization of risk by multivariate nonlinear regression analysis of each influencing factor of the roof fall and rib spalling accident; Gul et al.^13^ quantitatively assessed the risk of underground mining in copper-zinc mines based on Pythagorean fuzzy environment. These studies provide some theoretical and technical support for mine risk management and disaster prevention and control, however, most of the existing assessment methods are studies for single-hazard risks, which are mostly applied to simple systems. In contrast, underground mine systems are under the threat of multi-hazard risk. Under the conditions of mining disturbance, multi-hazard underground mines are subject to the interaction of complex factors, and the possibility of accidents is extremely high, the probability of disaster occurrence varies, and the consequences caused vary widely, and the characteristics and disaster-causing mechanisms of various disasters are also different. Single-hazard analysis cannot realize the unified measurement of risk and fails to reflect the comprehensive risk of underground mines. For complex system risk assessment, there is a lack of using an assessment method that is applied to multiple local systems and reflects the overall system risk. In addition, the multiple coexistence of hazards due to natural hazards or human influences makes the production process of multi-hazard underground mines face more severe tests of safety problems. It is highly characteristic to conduct research on multi-hazard risk assessment and prevention and control in mines, but most of the current research on multi-hazard risk is for natural hazards^14,15^, and there are relatively few studies on multi-hazard risk in mines. To assess the earthquake–landslide–debris flow hazard chain risk in Lanzhou, China, Lyu et al.^16^ used AHP to assess various geological risks, and then used geographic information systems (GIS) to map the spatial distribution of the earthquake–landslide–debris flow hazard chain risk. In addition, Kaur et al.^17^ and Gill et al.^18^ both adopted regional multi-hazard maps based on AHP and GIS to characterize regional multi-hazard risks. Pouyan et al.^19^ used three state-of-the-art machine learning techniques (boosted regression tree, random forest, and support vector machine) to produce a multi-hazard (MHR) map illustrating areas susceptible to fooding, gully erosion, forest fires, and earthquakes in Kohgiluyeh and Boyer-Ahmad Province, Iran. Pourghasemi et al.^20^ developed a multi-hazard probability map for three hazards (i.e. landslides, floods, and earthquakes) for the management of hazard-prone areas in Lorestan Province, Iran, using anew ensemble model named SWARA-ANFIS-GWO. The risk analysis method of multi-hazard risk map based on GIS is the mainstream of current multi-hazard risk research, but this method often requires a large amount of historical disaster data and a high-resolution regional overview map, which is suitable for analyzing the risk of natural hazards with a large impact area.

Realizing the unified measure of multi-hazard risk is the key to multi-hazard risk analysis. Multi-hazard risk lack unified metrics due to different mechanisms, nature, intensity, and risk exposure elements, and the risk aggregation model lacks universality, which makes it difficult to achieve effective multi-hazard risk cooperative management and control. To this end, combining risk assessment science, disaster system theory, and multi-hazard superimposed risk methods, statistical analysis of non-coal underground mine accidents is first conducted to determine the types of major disasters in non-coal underground mines. By standardizing the classification scheme of frequency and intensity thresholds of different disaster types, thus forming semi-quantitative categories or ranges, comparison between different disaster types is realized. Then a comprehensive risk assessment of multi-hazard sources in non-coal underground mines is conducted through regional risk aggregation, and corresponding risk synergy control strategies are proposed. Our research focuses on the first multi-hazard risk model, which addresses multi-hazard risk in mining regions, ignores any type of disaster chains, and forms semi-quantitative risk intervals or risk categories by standardizing a grading scheme for frequency and intensity thresholds for different types of hazards, thus allowing comparison between different hazard types and regional aggregation of multi-hazard risk to form a spatially oriented multi-hazard The integrated risk assessment model can provide both support for macro policy formulation and guidance for specific spatial layout.

“Statistical analysis of multi-hazard accident characteristics” presents a statistical analysis of accidents in non-coal underground mines, identifies the types of multi-hazard risk in non-coal underground mines, and proposes a multi-hazard risk assessment framework for non-coal underground mines. “Characterization of multi-hazard risk in non-coal underground mines” proposes a multi-hazard risk characterization model to achieve a unified measure of multi-hazard risk and regional risk aggregation. “Multi-hazard risk collaborative control mode based on 'PDCA'” proposes a collaborative multi-hazard risk control strategy. “Case study” validates the model using a metal underground mine as an example. “Discussion and future work” provides a discussion and analysis of the results and an outlook for future research. The conclusions drawn from this study are summarized in “Conclusions”.

## Statistical analysis of multi-hazard accident characteristics

**Figure 1:**
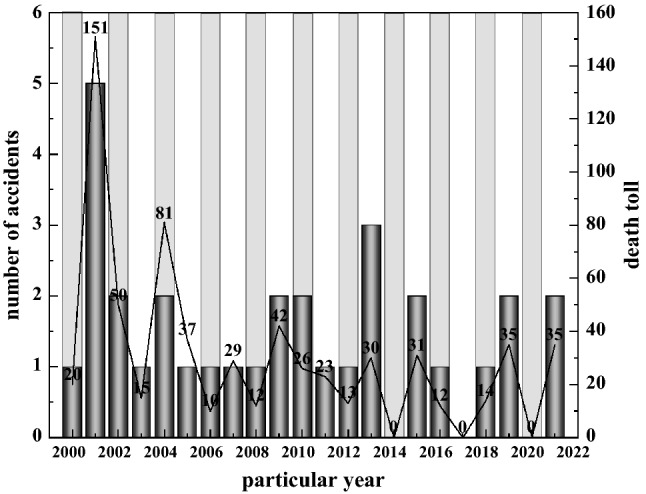
Statistics of major accidents in non-coal underground mines over the years.

A statistical analysis of accidents in non-coal underground mines in China^21^ showed that 31 major and especially major accidents occurred in non-coal underground mines during 2000–2021 (especially major accidents are accidents causing more than 30 deaths, or more than 100 serious injuries, or direct economic losses of more than 100 million yuan; major accidents are accidents causing more than 10 people and less than 30 a major accident is an accident that causes more than 10 people or less than 30 deaths, or more than 50 people or less than 100 serious injuries, or more than 50 million yuan or less than 100 million yuan in direct economic losses)^22^, including 4 especially major accidents and 27 major accidents, resulting in a total of 642 deaths, as shown in Fig. 1.

As can be seen from Fig. 1, the number of major accidents in underground mines in China over the past 20 years has maintained a steady trend, with the number of fatalities showing a slow downward trend, and major accidents basically occurring every year, with only 2014, 2017 and 2020 not having major accidents, indicating that major accidents in underground mines have not been completely curbed.

**Figure 2:**
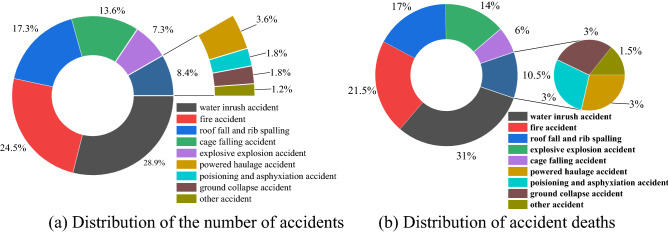
Distribution of the number of accidents and deaths of different accident types. (**a**) Distribution of the number of accidents. (**b**) Distribution of accident deaths.

The accident types of non-coal underground mines were statistically analyzed, and the distribution pattern of casualties of various accident types in non-coal underground mines was obtained, as shown in Fig. 2.

The time, place, cause and consequence of a single accident are uncertain, which shows that the prevention of accidents is somewhat difficult. But the randomness of accidents also follows the statistical law in a certain range. The results of statistical analysis of accidents in non-coal underground mines show that there is a certain regularity in the types of accidents, accident sites and accident times in non-coal underground mines. As can be seen from Fig. 2, the major accident types in non-coal underground mines are mainly water inrush, fire, roof fall and rib spalling, cage falling, etc. If the above accidents occur, it is likely to cause serious casualties and economic losses. The number of accidents and fatalities accounted for 70% and 78% of the total number of accidents of three types, including water inrush, fire and roof fall and rib spalling accident, and one particularly serious accident occurred. For this reason, we determine the major risk types of non-coal underground mines, such as "cage falling, roof fall and rib spalling, fire, powered haulage, water inrush" and other multi-hazard risk.

**Figure 3:**
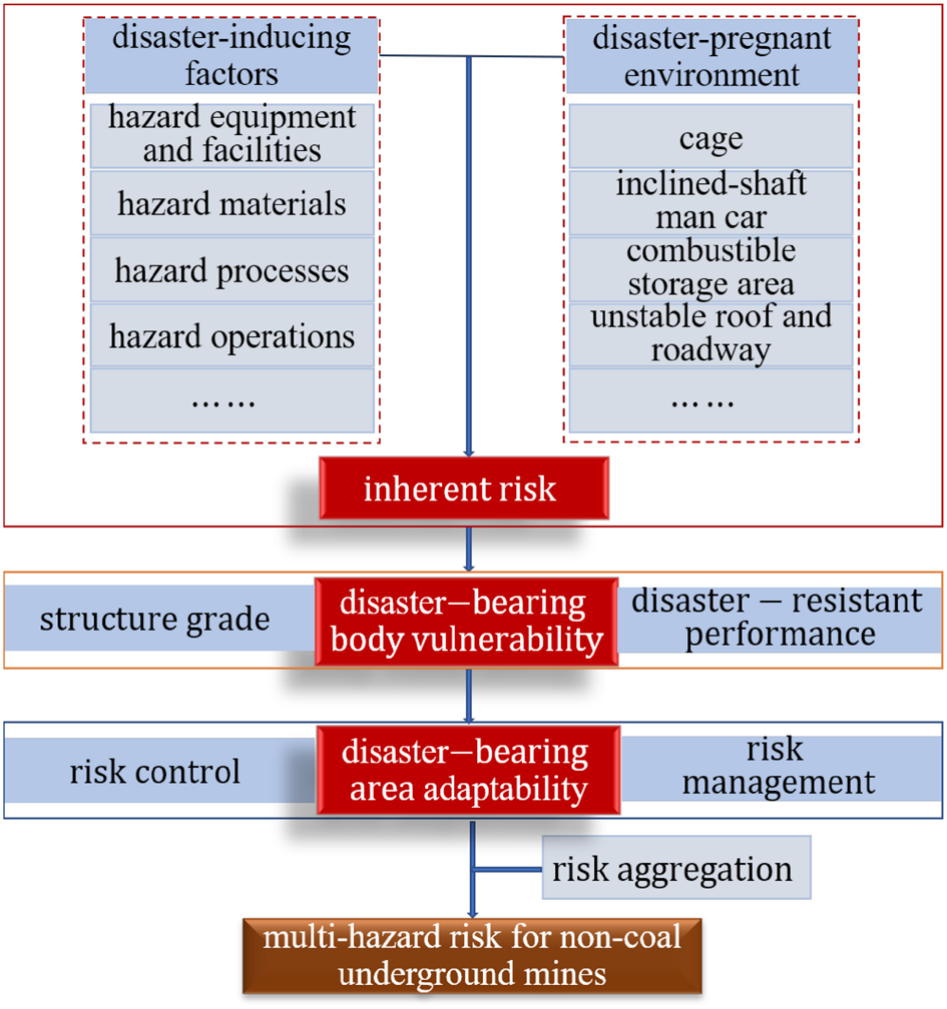
Risk assessment framework for multi-hazard sources in non-coal underground mines.

Safety risk evaluation of non-coal underground mines with multi-hazard is a composite process, and targeted evaluation elements should be selected according to different needs at different stages and refined into specific indicators for systematic study. According to the definition of risk by UNEDR^23^, Risk = Hazard × Vulnerability. On this basis, a multi-hazard risk analysis and assessment framework for non-coal underground mines based on the inherent hazards of the underground mine system (equipment, facilities, articles, processes, operations and sites), the vulnerability of the disaster-bearing body (structural strength level of the underground mine system or facilities, and disaster resistance), and the adaptability of the disaster-bearing area (risk control and risk management capability, early warning capability, emergency response capability, disaster prevention and relief capability, and recovery capability) is proposed, as shown in Fig. 3.

## Characterization of multi-hazard risk in non-coal underground mines

As can be seen from Fig. 2, among the underground mine accidents, cage falling accidents, Powered haulage accidents, fire accidents, water inrush accidents, and roofing and roof fall and rib spalling accidents occur with high frequency and serious consequences. In order to systematically evaluate the multi-hazard risk of non-coal underground mines, based on the system risk perception and system safety attribute index analysis, we systematically and comprehensively identify the risk of multi-hazard sources in non-coal underground mines based on five major hazards, such as "cage falling, roof fall and rib spalling, fire, power haulage and water inrush", and explore the multi-hazard risk description and quantification measures applicable to complex systems in non-coal underground mines. Based on the risk index method to characterize the inherent risk of multi-hazard risk in non-coal underground mines, coupling the vulnerability of the hazard-bearing body and the adaptability of the hazard-bearing area, a space-oriented multi-hazard risk characterization model is formed through regional risk aggregation.

### Risk characterization and measurement of inherent risk at non-coal underground mine risk points

The inherent risks of non-coal underground mines are the essential risks that exist within the system itself, which is the combination of factors within the system and the operators that have an impact, determined by its own properties and the relatively stable objective environment in which it is located.

Comparability of modeling results is the core problem of multi-hazard risk analysis, for which disaster risk indicators are divided into two levels: conceptual level and technical level, with the aim of transforming technically oriented risk concepts into quantifiable technical indicators and implementing risk distribution in space. Risk indicators at the conceptual level need to comprehensively characterize disaster risks, and five aspects, including hazardous equipment and facilities, hazardous materials, hazardous processes, hazardous operations, and hazardous places, are used as risk indicators at the conceptual level. The risk indicators at the technical level should be a refinement of the risk indicators at the conceptual level, and express the probable consequences of a disaster in a form that can be quantified in space. Finally, the inherent risk is characterized by the risk index method. Finally, the inherent risk of each risk point is characterized by the risk index method.

Characterization of indicators at the conceptual level in the cage falling accident risk point.

1. Hazardous equipment and facilities (*h*_*s*_)—Cage lifting system. Measured by the level of intrinsic safety of the manned cage lifting system.
2. Hazardous places (*E*)—Cage. Measured by the exposure risk index of the personnel working inside the cage and in cage maintenance.
3. Hazardous materials (*M*)—Material and people in the cage. Measured by the potential energy properties of the material and people in the cage.
4. Hazardous processes (*K*_1_)—Measured by the failure rate of monitoring facilities such as cage wire rope online monitoring and video monitoring facilities.
5. Hazardous operations (*K*_2_)—Measured by the type of high-risk operations involved in the cage lifting system.

The technical layer indicators and measurement scheme for the risk point of cage falling accident are shown in Table 1.

**Table 1:** Cage fall accident risk point.

Inherent risk	Quantitative indicators	Characteristic indicators	Quantitative value
*hs*	Hazard isolation	No hazard isolation measures	1.0
	Fault safe	Reliable protective devices and anti-dropping device	1.2
		The anti-dropping device failure, with reliable protection device	1.4
	Fault risk	The protection device fails, with anti-dropping device	1.3
		Failure of protective devices and anti-dropping device	1.7
*E*	Number of people exposed at risk point (P)	100 ≤ P	9
		30 < P ≤ 99	7
		10 < P ≤ 29	5
		3 < P ≤ 9	3
		0 ≤ P ≤ 2	1
M	Characterized by the classification result of inclined length *w* of shaft depth	*w* < 250	1
		250 ≤ *w* < 500	3
		500 ≤ *w* < 750	5
		750 ≤ w < 1000	7
		1000 ≤ *w*	9
*K*_1_	Wire rope on-line monitoring	Monitoring and control facilities failure rate *l*	1 + *l*
	Video surveillance facility		
*K*_2_	Equipment maintenance operation	Number of high-risk types of operations involved in cage lifting systems t	1 + 0.05t
	Elevator operation		
	Safety inspection operation		
	Hoist operation		

Characterization of indicators at the conceptual level in the powered haulage accident risk point.

1. Hazardous equipment and facilities (*h*_*s*_)—Inclined-shaft man car lifting system. Measured by the level of intrinsic safety of the inclined-shaft man car lifting system.
2. Hazardous places (*E*)—Inclined-shaft man car. Measured by the exposure risk index of the personnel working within the range of the inclined-shaft man car and inside the inclined-shaft man car.
3. Hazardous materials (*M*)—Vertical depth. Measured by the potential energy properties of the material and people in the inclined-shaft man car.
4. Hazardous processes (*K*_1_)—Measured by the failure rate of monitoring facilities such as inclined-shaft wire rope online monitoring and video monitoring facilities.
5. Hazardous operations (*K*_2_)—Measured by the type of high-risk operations involved in the inclined-shaft man car lifting system.

The technical layer indicators and measurement scheme for the risk point of powered haulage accident are shown in Table 2.

**Table 2:** Powered haulage accident risk point.

Inherent risk	Quantitative indicators		Characteristic indicators	Quantitative value
*hs*	Hazard isolation		Non-hazard isolation measures	1.0
	Fault safe	Fail safe	1.2	1.2
		Fail risk	1.4	1.4
	Fault risk	Fail safe	1.3	1.3
		Fail risk	1.7	1.7
*E*	Number of people exposed at risk point (P)		100 ≤ P	9
			30 < P ≤ 99	7
			10 < P ≤ 29	5
			3 < P ≤ 9	3
			0 ≤ P ≤ 2	1
M	Characterized by the classification result of inclined length w of shaft depth		*w* < 250	1
			250 ≤ *w* < 500	3
			500 ≤ *w* < 750	5
			750 ≤ w < 1000	7
			1000 ≤ *w*	9
*K*_1_	Wire rope on-line monitoring		Monitoring and control facilities failure rate *l*	1 + *l*
	Video surveillance facility			
*K*_2_	Equipment maintenance operation		Number of high-risk types of operations involved in powered haulage system	1 + 0.05t
	Motor vehicle driving operation			
	Safety inspection operation			
	Hoist operation			

Characterization of indicators at the conceptual level in the fire accident risk point.

1. Hazardous equipment and facilities (*h*_*s*_)—Fire prevention and extinguishing facilities. Measured by the setting of mine fire prevention and extinguishing facilities.
2. Hazardous places (*E*)—Combustible storage area. Measured by the exposure risk index for operators in the middle section of the combustible storage area.
3. Hazardous materials (*M*)—Combustible material. Measured by the danger of combustible materials.
4. Hazardous processes (*K*_1_)—Measured by the failure rate of monitoring and monitoring facilities such as toxic and hazardous gas monitoring (CO, NO_2_, etc.) and temperature alarm monitoring facilities.
5. Hazardous operations (*K*_2_)—Measured by the type of high-risk operations involved in the fire accident risk point.

The technical layer indicators and measurement scheme for the risk point of fire accident are shown in Table 3.

**Table 3:** Fire accident risk point.

Inherent risk	Quantitative indicators		Characteristic indicators		Quantitative value
*hs*	Types of fire prevention and extinguishing facilities *n*		fire extinguisher		1.7–0.175*n*
			fire hydrants		
			automatic fire-extinguishing installation		
			self-rescuer		
*E*	Exposure risk index of workers in the middle of combustible storage area		Number of people exposed at risk point (P)	100 ≤ P	9
				30 < P ≤ 99	7
				10 < P ≤ 29	5
				3 < P ≤ 9	3
				0 ≤ P ≤ 2	1
*M*	Dangers of combustible materials	*m* = *β*_1_ × (*q*_1/_*Q*_1_) + *β*_2_ × (*q*_2/_*Q*_2_) + $\cdots$+*β*_*n*_ × (*q*_*n*/_*Q*_*n*_)	*m* < 1		1
			1 ≤ *m* < 10		3
			10 ≤ *w* < 50		5
			50 ≤ w < 100		7
			100 ≤ *w*		9
*K*_1_	Toxic and harmful gas monitoring		Monitoring and control facilities failure rate *l*		1 + *l*
	Temperature monitoring				
*K*_2_	Temporary electricity operation		Number of high-risk types of operations involved in powered haulage system		1 + 0.05t
	Ignition operation				
	Welding gas cylinder operation				
	Welding and thermal cutting operations				
	Safety inspection operation				
	Electrical operation				
	Ventilation operation				

where *Q*_1_, *Q*_2_ $,\cdots$ *Q*_*n*_ are the critical quantities; *q*_1_, *q*_2,_$\cdots$ *q*_*n*_ are the actual storage quantities; and the correction factor *β* takes the value of 1.

Characterization of indicators at the conceptual level in the mine water inrush accident risk point^24^.

1. Hazardous equipment and facilities (*h*_*s*_)—waterproofing and drainage facilities. Measured by the setting of mine waterproofing and drainage facilities.
2. Hazardous places (*E*)—The lowest middle section of the mine. Measured by the exposure risk index of the workers in the lowest middle section of the mine.
3. Hazardous materials (*M*)—Mine water filling source. Measured by the complexity of hydrogeological conditions at the mine area.
4. Hazardous processes (*K*_1_)—Measured by the failure rate of monitoring facilities such as mine water inflow monitoring, precipitation monitoring, and video monitoring facilities at the water exploration operation surface.
5. Hazardous operations (*K*_2_)—Measured by the type of high-risk operations involved in the mine water inrush accident risk point.

The technical layer indicators and measurement scheme for the risk point of mine water inrush accident are shown in Table 4.

**Table 4:** Mine water inrush accident risk point.

Inherent risk	Quantitative indicators	Characteristic indicators		Quantitative value
*hs*	Types of waterproof and drainage facilities (*n*)	Waterproof gate		1 + 0.175*n*
		Grouting sealing		
		Hydrophobic decompression		
		Drainage system		
		Stream closure management		
*E*	Exposure risk index for lowest mid-level workers	Number of people exposed at risk point (P)	100 ≤ P	9
			30 < P ≤ 99	7
			10 < P ≤ 29	5
			3 < P ≤ 9	3
			0 ≤ P ≤ 2	1
*M*	The complexity of hydrogeological conditions in mining area	Simple		2
		Medium		5
		Complex		8
*K*_1_	Water inflow monitoring	Monitoring and control facilities failure rate *l*		1 + *l*
	Precipitation monitoring			
	Video surveillance of water exploration face			
*K*_2_	Water exploration and drainage operation	Number of high-risk types of operations involved in powered haulage system		1 + 0.05t
	Safety inspection operation			
	Drainage operation			

Characterization of indicators at the conceptual level in the roof fall and rib spalling accident risk point^12^.

1. Hazardous equipment and facilities (*h*_*s*_)—Roof of mined-out area. Measured by the rock quality index grade of the roof of mined-out area.
2. Hazardous places (*E*)—Unstable roof roadway and goaf. Measured by the exposure risk index of the workers in the unstable roof roadway and goaf.
3. Hazardous materials (*M*)—Empty roof. Measured by the degree of treatment of goaf.
4. Hazardous processes (*K*_1_) —Measured by the failure rate of ground pressure monitoring and monitoring facilities such as roof subsidence monitoring and surface subsidence monitoring in mining areas.
5. Hazardous operations (*K*_2_)—Measured by the type of high-risk operations involved in the roof fall and rib spalling accident risk point accident risk point.

The technical layer indicators and measurement scheme for the risk point of roof fall and rib spalling accident are shown in Table 5.

**Table 5:** Roof fall and rib spalling accident risk point.

Inherent risk	Quantitative indicators	Characteristic indicators		Quantitative value
*hs*	Roof rock mass quality index	Rock mass basic quality (BQ)	550 ≤ BQ	1 + 0.175*n*
			451 ≤ BQ < 550	
			351 ≤ BQ < 450	
			251 ≤ BQ < 350	
			BQ ≤ 250	
*E*	Exposure risk index of operators in unstable roof roadway and goaf	Number of people exposed at risk point (P)	100 ≤ P	9
			30 < P ≤ 99	7
			10 < P ≤ 29	5
			3 < P ≤ 9	3
			0 ≤ P ≤ 2	1
M	Degree of goaf treatment	Complete treatment		1
		local treatment		6.3
		untreated		9
		Continuous goaf volume > 1 × 10^6^m^3^		9
*K*_1_	Roof subsidence monitoring	Monitoring and control facilities failure rate *l*		1 + *l*
	Surface subsidence monitoring			
*K*_2_	Number of high-risk types of operations involved in powered haulage system	Safety inspection operation		1 + 0.05t
		Pillar operation		
		blasting operation		

The inherent risk index (*Hi*) of each accident risk point in the non-coal underground mining system is.

1 $${H}_{i}={h}_{s}ME{K}_{1}{K}_{2}$$

where *Hi* is the inherent risk index of the *i*th risk point, *h*_*s*_ is the hazard coefficient of hazardous equipment and facilities, *M* is the hazard coefficient of the hazardous materials, *E* is the hazard coefficient of the hazardous places, *K*_*1*_ is the hazard coefficient for hazardous processes, *K*_*2*_ is the hazard coefficient for hazardous operations.

### Characterization of the vulnerability of non-coal underground mine systems

The vulnerability of non-coal underground mines refers to the ability of the safety protection system of underground mines to maintain its main safety features and functions without being significantly affected when facing the impact of external accidents. Define the vulnerability of non-coal underground mine systems as^25^.

2 $$V={r}_{1}{x}_{1}^{^{\prime}}+{r}_{2}{x}_{2}^{^{\prime}}+{r}_{3}{x}_{3}^{^{\prime}}+{r}_{4}{x}_{4}^{^{\prime}}+{r}_{5}{x}_{5}^{^{\prime}}$$

where *V* is the vulnerability of the non-coal underground mine system, $V\in [\mathrm{0,1}]$, $r_{1} ,\,\,r_{2} ,\,\,r_{3} ,\,\,r_{4} ,\,\,r_{5}$ are the weights of equipment and facilities, places, materials, processes, operations in the probability of accidents, derived from the AHP method and $x_{1}^{^{\prime}} ,\,\,x_{2}^{^{\prime}} ,\,\,x_{3}^{^{\prime}} ,\,\,x_{4}^{^{\prime}} ,\,\,x_{5}^{^{\prime}}$ respectively for equipment and facilities, places, materials, processes, operations of the degree of hazard.

3 $$ r_{1} + r_{2} + r_{3} + r_{4} + r_{5} = 1,\quad x_{i}^{^{\prime}} = 1 - x_{i} . $$

Substituting this into Eq. (2) yields.

4 $$V=1-{r}_{1}{x}_{1}-{r}_{2}{x}_{2}-{r}_{3}{x}_{3}-{r}_{4}{x}_{4}-{r}_{5}{x}_{5}$$

where $x_{1} ,\,\,x_{2} ,\,\,x_{3} ,\,\,x_{4} ,\,\,x_{5}$ are equipment and facilities, places, materials, processes, operations safety degree, determined by the measurement results of the risk indicators inherent in each risk point.

**Figure 4:**
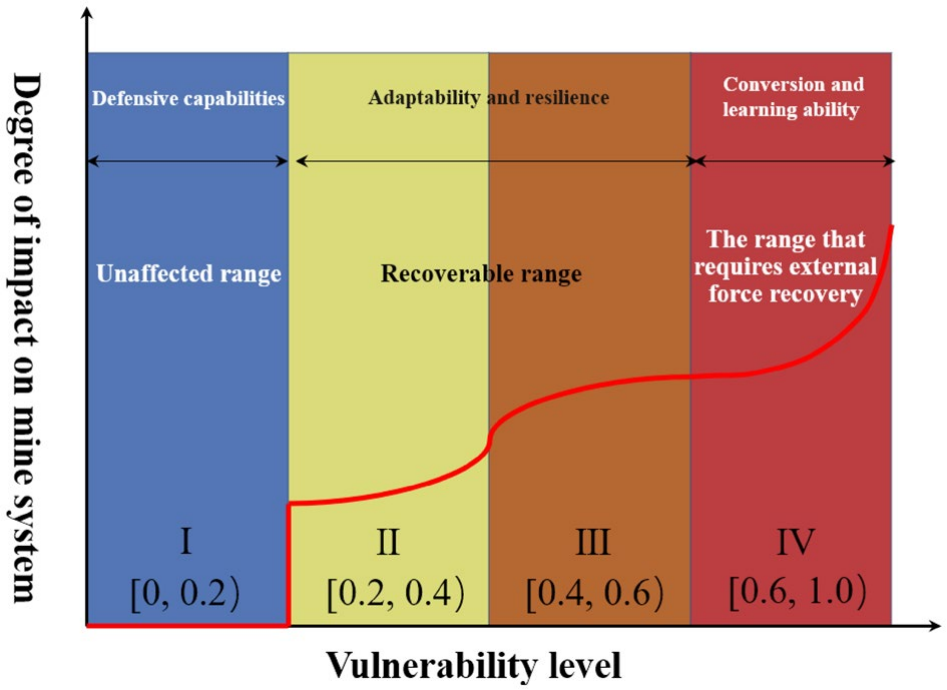
Vulnerability classification model for non-coal underground mine systems.

Underground mines have certain adaptability in accident and disaster environment. When subjected to a minor accidental impact, the underground mine system can absorb the impact force, when subjected to a moderate accidental intensity, it can attenuate the impact force, and when subjected to a larger intensity impact, it can withstand and quickly recover by external means, which also reflects the size of the vulnerability of the non-coal underground mine system, and its corresponding stages of development are shown in Fig. 4. According to the vulnerability results of the non-coal underground mine system, it is divided into four levels: I, II, III and IV, which correspond to the four stages of vulnerability levels in Fig. 4.

### Characterization of disaster-bearing area adaptability

Standardized management is implemented for the enterprise from eight elements: target responsibility, institutionalized management, education and training, on-site management, safety risk control and hidden danger investigation and treatment, emergency management, accident management, and continuous improvement. The effective production safety management system can reduce the frequency of accidents. The standardized production safety assessment method of enterprises is used to characterize the adaptability index of the disaster-bearing area and measure the level of safety risk control of enterprises to reflect the adaptability of the disaster-bearing area of non-coal underground mining system. According to the scoring method for safety production standardization of non-coal underground mines, the full score of the initial safety production standardization grade is 100. Take the reciprocal of the standardized score rate as the adaptability index of the disaster-bearing area, and the adaptability index of the disaster-bearing area is.

5 $$ G = {1}00/v $$

where *G* is the index of adaptability to the disaster-bearing area and *v* is the evaluation score of safety production standardization.

### Multi-hazard risk aggregation and classification of non-coal underground mine

The inherent risk index *H* of each risk point, the vulnerability index *V* of the hazard-bearing body and the adaptive risk index *G* of the hazard-bearing area are aggregated to obtain the safety risk *R*_*i*_ of single hazard.

6 $$ R_{i} = HVG $$

The Nemerow pollution index is a weighted multi-factor environmental quality index that takes into account the extreme values or highlights the maximum values^26^. Considering the influence of the most serious factors within the non-coal underground mine system and avoiding the influence of subjective factors in the weight coefficients, the Nemero pollution index is introduced into the multi-hazard risk aggregation, and the Nemerow pollution index method is used for the regional aggregation of risks in non-coal mine systems.

According to the safety risk *R*_*i*_ of each risk point of non-coal underground mines, from which the maximum risk value *max*(*R*_*i*_) and the average value *ave*(*R*_*i*_) are determined, the regional risk aggregation according to the Nemerow pollution index, the regional multi-hazard aggregated risk (*R*_*C*_) of non-coal underground mines is.

7 $$ R_{C} = \sqrt {\frac{{max(R_{i} )^{2} + ave(R_{i} )^{2} }}{2}} $$

where, *R*_*C*_ is the multi-hazard area aggregated risk value of non-coal underground mine, *R*_*i*_ is the single-hazard risk value, *max*(*R*_*i*_) is the largest risk value of each risk point in the non-coal underground mine system and *ave*(*R*_*i*_) is the average risk values of each risk point in the non-coal underground mine system.

The multi-hazard risk in the non-coal underground mine area is divided into four levels, i.e., level I (major risk), level II (greater risk), level III (general risk), level IV (low risk). The four colors of "red", "orange", "yellow" and "blue" are used as risk warning levels. The grading results are shown in Table 6.

**Table 6:**
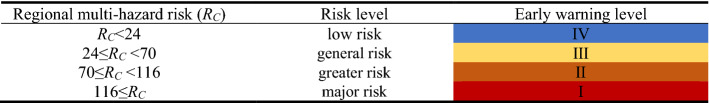
Classification of regional multi-hazard risk levels.

The key to risk classification lies in the determination of the classification standard, but it should be noted that the risk classification standard should not be absolutely constant, the so-called low risk and major risk are only artificially defined at present, and the risk classification standard needs to be continuously optimized in conjunction with the increasing assessment samples.

## Multi-hazard risk collaborative control mode based on 'PDCA'

**Figure 5:**
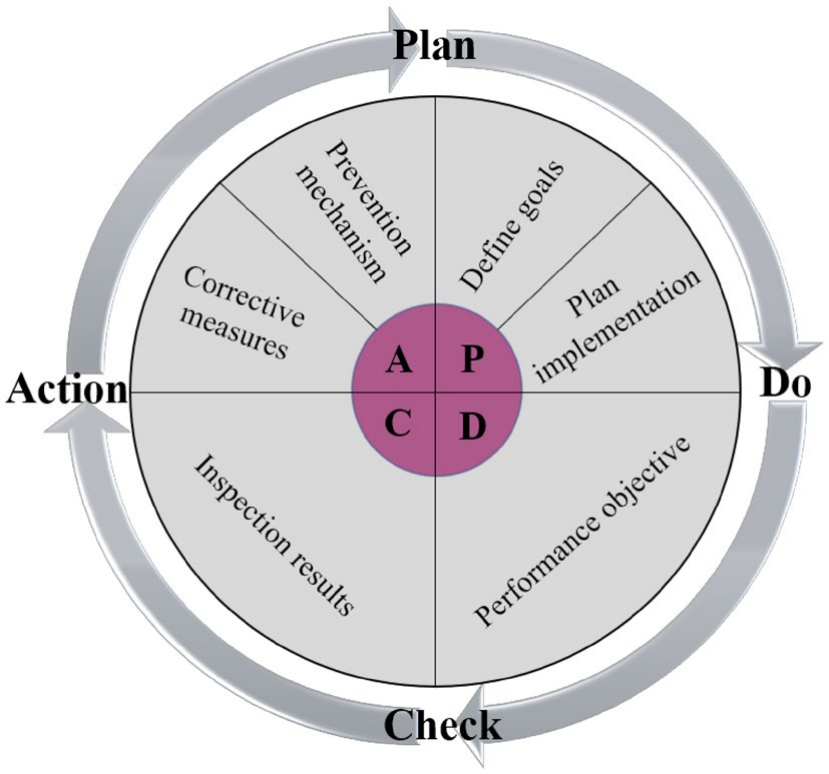
The process of 'PDCA' cycle.

'PDCA' is a combination of the initials Plan, Do, Check and Action. It is the scientific procedure that should be followed for total quality management, no matter which work is inseparable from the 'PDCA' cycle, each work needs to go through the four stages of planning, implementing the plan, checking the plan, making adjustment to the plan and continuous improvement. The basic cycle of 'PDCA' is shown in Fig. 5.

**Figure 6:**
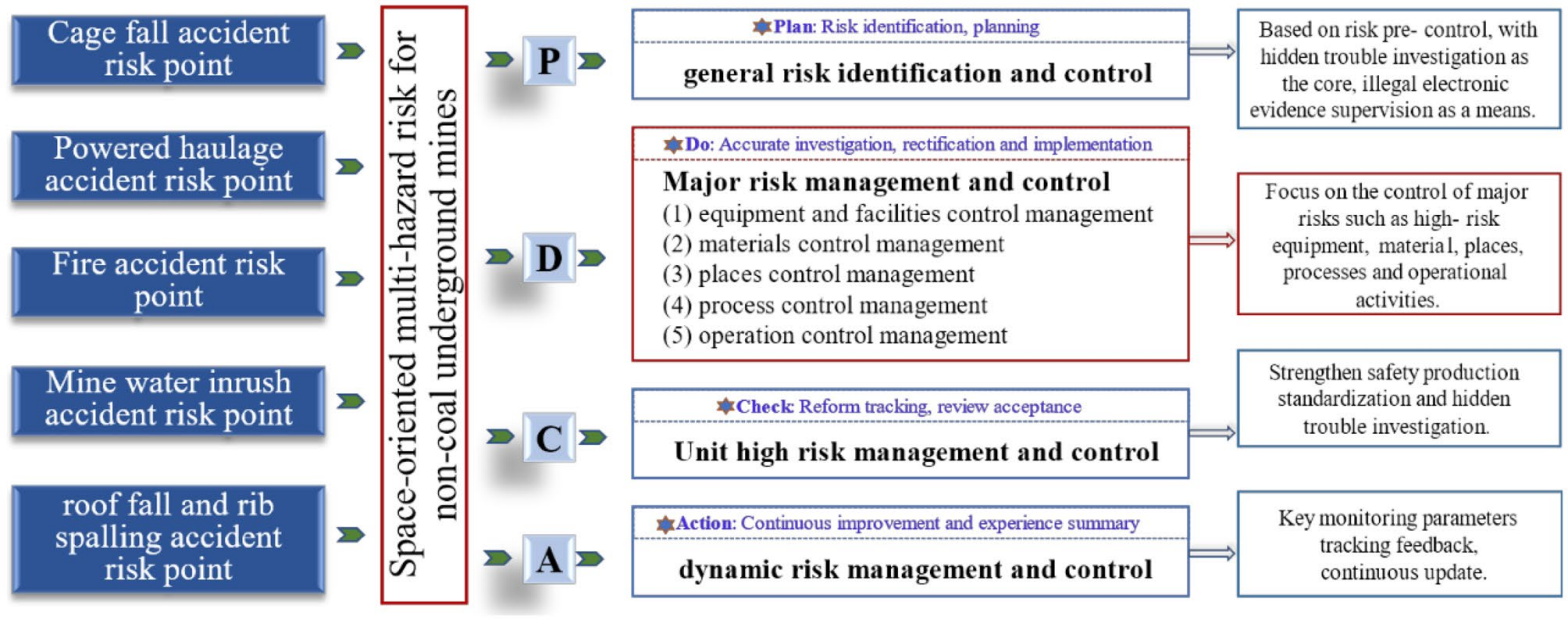
'PDCA' closed-loop control model for multi-hazard risk in non-coal underground mines.

Brown^27^ states, that engineering science has evolved enough to deal with technical risks, and that the more easily overlooked risks are non-technical ones, most of which are the result of poor management. To this end, the 'PDCA' closed-loop control model of non-coal underground mines risk is established, highlighting the risk management of hazardous operations, hazardous processes, hazardous equipment and facilities, hazardous materials and hazardous places, and the safety risk control system of source identification, classification control, process control, continuous improvement and full participation is constructed, as shown in Fig. 6.

1. Taking risk pre-control as the core, hidden danger investigation as the basis, and illegal electronic evidence supervision as the means, the 'PDCA' closed-loop management operation model of tailings pond is established. Rely on the scientific assessment and evaluation mechanism to promote its effective operation, planning risk prevention and control measures, implementing tracking feedback, and continuously updating risk dynamics and prevention and control process.
2. Implement risk classification management and control, especially the major risks of non-coal underground mines, focus on high-risk equipment and facilities, material, processes, operation, places, strengthen dynamic risk management and control, and realize prevention and control.
  - Management and control of hazardous equipment and facilities. High-risk equipment and facilities should be implemented in strict accordance with the design and safety procedures to improve the intrinsic safety of equipment and facilities. The design and construction of underground mining system in non-coal mines must meet the requirements of national laws, regulations and standard specifications.
  - Management and control of hazardous materials. For cages, explosives and unstable roadway roofs that may lead to major accidents, the parameters of hazardous materials should be strictly controlled according to relevant safety standards and design requirements, and daily detection, testing and maintenance and other management should be done well.
  - Management and control of hazardous places. Personnel exposure in hazardous places should be reduced, measures of 'automatic personnel reduction and mechanized replacement' should be taken, and remote inspection technology should be promoted. The entry of personnel such as temporary operators, supervisors, nearby residents and so on into the mine area, will also increase the risk of the mine, enterprises should strengthen the monitoring of mobile personnel.
  - Management and control of hazardous processes. Ensure the normal operation of data and transmission of non-coal mine safety online monitoring system, improve the reliability of key monitoring data, and complete the safety online monitoring recovery as soon as possible in case of failure.
  - Management and control of hazardous operations. Practitioners shall receive education and training on work safety, master the knowledge of work safety required for their own work, correctly cognitive the post safety risks and relevant control measures, enhance accident prevention and emergency response capabilities, the implementation of licensed work, and do a good job of safety education before starting work.
3. Improve the safety management level of enterprise. Strengthen the risk management and control of non-coal underground mines, establish the intelligent identification system of hidden dangers and violations, strengthen the investigation and reporting of hidden dangers, make dynamic adjustment according to the actual state of the enterprise's safety management level, and make it timely reflect the actual risk control level of the enterprise.
4. Strengthen risk dynamic management and control. Based on the basic dynamic management information of non-coal mine, geological disaster information to make timely response measures, improve the real-time and validity of risk dynamic index data, and avoid data distortion. Build a big data support platform to strengthen the linkage of meteorological and geological disaster information.

## Case study

Taking a large scale ferrous metallurgical underground mine in Hubei Province, China as an example, the regional multi-hazard risk assessment is carried out for the risk points of cage falling accident, fire accident, water inrush accident and roof fall and rib spalling accident involved in this underground mine, and corresponding risk control strategies are proposed to verify the feasibility and reliability of the model.

### Mine overview

The main product of this iron ore is magnetite, with an annual capacity of 1.3 million tons. The surrounding rocks of the ore body roof mainly include skarn, marble, dolomitic marble and skarn diorite, while the surrounding rocks of the ore body floor mainly include skarn, skarn diorite and biotite pyroxene diorite. The orebody belongs to inclined ~ steeply inclined and medium thick ~ thick ore body. The stability of the ore is moderately stable ~ stable, and the surrounding rock of the roof and floor is moderately stable ~ stable. The ore is dominated by massive magnetite-rich and mixed-rich.

The mine adopts shaft development, and the main mining sections are − 50 m, − 110 m and − 170 m. The ore is mainly lifted from phase I main shaft, phase II main shaft and phase III main shaft. The design lifting capacity of phase I main shaft is 700,000 tons, phase II main shaft is 300,000 tons, and phase III main shaft is 450,000 tons. Underground transportation adopts electric locomotive to transport ore.

The mining method is sublevel open stope and subsequent filling method, Simba 1252 medium and deep hole rock drilling jumbo drilling, granular ANFO explosive and non-electric blasting system blasting, 3m^3^ electric or diesel scraper concentrated at the bottom of the stope for ore extraction, and after the stope stoping is completed, full tailings filling is used for cemented filling of the goaf.

Diagonal mechanical forced ventilation is adopted, the return air shaft is equipped with exhaust equipment, and local fans are used for auxiliary ventilation in local areas with poor ventilation. There is a ventilation monitoring system to monitor the air volume, wind speed and wind quality of each mining area. In addition, the mine implements wet rock drilling, and the crushing base station is equipped with a closed dust hood for dust removal.

### Multi-hazard risk measurement

(1) Risk assessment of cage falling accident risk point.

The safety protection device of the auxiliary shaft cage of the mine is normal, the steel wire rope braking system and the hoist system are normal, and reliable overwinding protection devices and fall arresters are set. Hazard index of hazardous equipment and facilities is *h*_*s*_ = 1.2.

The depth of the auxiliary shaft in the mining is 190 m, and the hazard index of hazardous substances *M* = 1 is determined by the depth of the auxiliary shaft.

The working system of the mine is 3 shifts/day, 8 h per shift, 150 people in the morning shift, 60 people in the middle shift and 60 people in the evening shift in the mine. According to the maximum number of people in the cage is 48, the hazard index of hazardous places is determined to be *E*_1_ = 7.

The steel wire rope of the hoisting system has complete inspection records, but the steel wire rope online monitoring system has not been built. In addition, video monitoring facilities and other monitoring facilities are normal. Hazard index of hazardous processes *K*_1_ = 1.5.

The hazardous operations involved in the risk points of cage falling accidents include equipment maintenance operations, elevator operations, non-coal mine safety inspection operations, and non-coal mine hoist operations. The risk correction coefficient of hazardous operations *K*_2_ = 1.2.

Therefore, the inherent risk index *H*_1_ = 1.2 × 1 × 7 × 1.5 × 1.2 = 15.12 for the risk point of cage falling in the mine.

The system vulnerability of the cage falling accident of the mine *V* = 1–0.07–0.01–0.15–0.18–0.225 = 0.365.

The safety production standardization level of the underground mine is level I, taking *v* = 90, that is, the risk control index *G* = 1.11.

The risk of the risk point of the tank falling accident of the mine *R*_1_ = *GHV* = 6.13.

(2) Risk assessment of fire accident risk point.

The mine has obvious fire prevention signs and precautions. The duty room at the entrance of the auxiliary ramp is equipped with fire-fighting equipment, mainly including dry powder fire extinguishers, gas masks, etc. A certain amount of sand is stacked on the surface for fire extinguishing. Each trackless self-propelled equipment (including underground LHD, rock drilling jumbo, material truck, etc.) is equipped with fire extinguishing devices. Operators and temporary personnel must wear a self-rescuer when going down the shaft. However, the mine is not equipped with automatic fire extinguishing facilities. Therefore, the hazard index of hazardous equipment and facilities is *h*_*s*_ = 1.175.

There is diesel fuel stored in the well, a 3 m^3^ tank in the bottom of the well yard and a 180L drum at the working face, with an oil hazard index of *M*_1_ = 1. Ore product is mainly magnetite, no spontaneous combustion tendency, ore hazard index *M*_2_ = 1. The cables and supports under the mine are non-flame-retardant materials, and the hazard index of non-flame-retardant materials *M*_3_ = 1. *M* = *max*(*M*_1_*,M*_2_*,M*_3_) = 1.

The maximum number of people working in a single shift under the mine is 150. Considering the anti-wind system and the number of people affected by a fire in the underground yard is calculated as 100, the hazard index of the hazardous places is determined as *E*_2_ = 9.

There are monitoring facilities such as toxic and hazardous gas detection devices and temperature alarms built under the mine, which are all operating normally and have good air quality. Hazard index of hazardous processes *K*_1_ = 1.

The hazardous operations involved in fire accident risk points include temporary power operation, fire work, pressure vessel operation, welding and thermal cutting operation, non-coal mine safety inspection operation, non-coal mine underground electrical operation, and mine ventilation operation. The risk correction coefficient of hazardous operations *K*_2_ = 1.35.

Therefore, the inherent risk index *H*_2_ = 1.175 × 1 × 9 × 1 × 1.35 = 14.28 for the risk point of cage falling in the mine.

The system vulnerability of the fire accident of the mine *V* = 1–0.069–0.011–0.2–0.15–0.203 = 0.567.

The risk control index *G* = 1.11.

The risk of the risk point of the fire accident of the mine *R*_2_ = *GHV* = 8.99.

(3) Risk assessment of mine water inrush accident risk point.

Surface waterproofing measures and facilities in the mine area include: interception and drainage ditches have been excavated around the mine area to effectively intercept the convergence of surface water from the surrounding areas to the collapse area; waterproofing inspections are organized and manned during the rainy season every year. The main waterproofing measures in the underground mine: water exploration work was carried out in the newly mined area, and waterproof doors were installed in each of the main production sections − 50 m, − 110 m and − 170 m at the bottom of the shaft yard; the water outlet points with a large amount of water gushing from the shaft were sealed; the main drainage equipment and facilities are nine 200D43 × 8 pumps with a head of 344 m and a flow rate of 280m^3^/h. Therefore, the hazard index of hazardous equipment and facilities is *h*_*s*_ = 1.14.

The hydrogeological conditions of the mine area belong to simple to moderately complex type; the maximum water inflow is 24430 m^3^/day, the normal water inflow is 3432 m^3^/day. The hazard index of hazardous materials *M* = 4.

The lowest middle section of the mine is − 170 m, the maximum number of workers in a single shift is about 30, and the hazard index of hazardous places is determined to be *E*_3_ = 9.

The surface of the mining area is equipped with precipitation monitoring, and the underground is equipped with monitoring facilities such as pit water inflow monitoring, which are operating normally. The mining area has completed the water exploration work in the newly opened area, and currently there is no water exploration work surface, and the water exploration work surface is monitored by video. Hazard index of hazardous processes *K*_1_ = 1.

The hazardous operations involved in the risk points of water inrush accidents are water exploration and drainage operations, mine safety inspection operations, and mine drainage operations in three categories, with the high-risk operation hazard correction factor *K*_2_ = 1.15.

Therefore, the inherent risk index *H*_3_ = 1.175 × 1 × 9 × 1 × 1.35 = 47.20 for the risk point of water inrush in the mine.

The system vulnerability of the water inrush accident of the mine *V* = 1–0.067–0.044–0.2–0.15–0.173 = 0.366.

The risk control index *G* = 1.11.

The risk of the risk point of the water inrush accident of the mine *R*_3_ = *GHV* = 19.0.

(4) Risk assessment of roof fall and rib spalling inrush accident risk point.

According to the engineering geological characteristics of rock discontinuity structural plane and structural body, the rocks in the mining area are divided into three engineering geological rock groups. Massive rock group: mainly thick to mega-thick layered limestone and granitic diorite porphyry, dominated by massive structure, with RQD value of 50–80%, which is a grade II rock body, and grade IV rock body at broken places. Loose structure rock group: It is mainly the surface quaternary strata and the loose rock formation in the fracture belt, belonging to class V rock mass. Therefore, the hazard index of hazardous equipment and facilities is *h*_*s*_ = 1.175.

At present, the mine adopts the filling method for mining, the goaf is treated completely, the roadway is supported by shotcrete-bolt mesh in the whole process, and the local unstable area is supported by I-steel support. The hazard index of hazardous materials *M* = 1.

The working system of the mine is 3 shifts/day, 8 h per shift, and 5 people per shift for a single stope work surface. High risk site hazard index *E*_4_ = 3.

Surface subsidence monitoring facilities have been built on the surface of the mine area, and some ground pressure activity areas have ground pressure monitoring facilities, and the monitoring data can be obtained normally. Hazard index of hazardous processes *K*_1_ = 1.25.

The hazardous operations involved in the risk point of roof fall and rib spalling accident are mine safety inspection operation, mine pillar operation, mine blasting operation 3 categories, high-risk operation hazard correction factor *K*_2_ = 1.15.

Therefore, the inherent risk index *H*_4_ = 1.175 × 1 × 3 × 1.25 × 1.15 = 5.07 for the risk point of roof fall and rib spalling in the mine.

The system vulnerability of the roof fall and rib spalling accident of the mine *V* = 1–0.069–0.011–0.067–0.188–0.173 = 0.492.

The risk control index *G* = 1.11.

The risk of the risk point of the roof fall and rib spalling accident of the mine *R*_4_ = *GHV* = 3.11.

The metal underground mine is under the greatest threat of water inrush accident, followed by fire accident, cage falling accident and roof fall and rib spalling accident.

Based on the Nemerow pollution index method for regional risk aggregation, the regional multi-hazard risk index of non-coal underground mine system is obtained, $R_{C} = \sqrt {19^{2} + 9.31^{2} /2}$ = 21.2. According to the safety risk classification standard of non-coal underground mines in Table 6, the safety risk level of the underground mine is level IV.

### Cooperative management and control strategy of multi-hazard risk

Risk control measures for the risk point of the roof fall and rib spalling accident.

1. Reduce the stress concentration zones as far as possible in the mining technical scheme;
2. Strengthen the roadway support work. Graded support should be implemented according to the stability of the rock mass, and the basis of graded support should be reliable, especially the broken section should be designed separately and should be supported in time.
3. Ground pressure monitoring measures are taken in necessary areas. Ground pressure monitoring is recommended in areas where stress is relatively concentrated.

Risk control measures for the risk point of the cage falling accident.

1. Recommends enhanced inspection of well tower structures, anti-over-coil devices, and loading and unloading facilities.
2. It is suggested to improve the inspection items, standards and methods of the daily checklist.

Risk control measures for the risk point of the mine water inrush accident.

1. A comprehensive analysis of mine water flow over the years to identify their patterns.
2. Revise and improve the emergency plan for underground waterproofing, and organize drills.

In addition, the future operation of the mine should continue to implement the safety management system, strengthen the daily inspection of safety facilities such as cages, combustible storage areas, drainage facilities and monitoring facilities, and deal with the problems found in a timely manner, and make the corresponding records and archiving work.

## Discussion and future work

The goal of disaster risk assessment is to provide a basis for the setting of comprehensive disaster prevention planning goals and lay the foundation for the planning of the overall spatial layout of disaster prevention by using historical disaster situations as the basis, reasonably predicting future development trends, comprehensively analyzing the potential impacts of multiple disasters on society, economy and environment, and combining disaster risks with spatial elements. Through accident statistics analysis of non-coal underground mines, it was found that non-coal underground mine systems are affected by multiple hazards and that risk can only be effectively reduced by considering and analyzing all relevant threats. However, risk assessment of multiple hazards poses an additional set of challenges compared to single-hazard analysis due to the different characteristics of the processes. Because of the need for comparability of single-hazard results, an equivalent method must be chosen to rank single risks and estimate the overall risk level. The inherent risks of various hazards are comprehensively identified, quantified and evaluated from five aspects, including hazardous equipment and facilities, hazardous materials, hazardous processes, hazardous operations and hazardous places, and then combined with the vulnerability of the disaster-bearing body and the adaptability of the disaster-bearing area, the unified measurement of multi-hazard risk is well realized and the comparability of risks is achieved. The core achievements of disaster risk assessment include two parts, one is the ranking of disaster levels based on risk quantitative evaluation, and the other is the comprehensive risk zoning reflecting the impact level and spatial distribution of multiple disasters. The hazard ranking determines the priority types of disasters to be responded to based on the analysis of the possibility of disaster occurrence. The comprehensive risk zoning map, on the other hand, clarifies the safety of different areas in space and provides a basis for the spatial layout and facility configuration for disaster prevention.

The interaction and spatial distribution analysis of multi-hazard risk are important research directions in the future. Due to the mutual cause and effect or homologous occurrence of natural disasters or social disasters, multi hazard disasters in non-coal underground mines may interact with each other, and multi-hazard disasters in the underground mining process may directly or indirectly trigger the occurrence of other hazards in the mining process, such as water inrush accidents and roof fall and rib spalling accidents caused by the collapse of the same goaf. Therefore, cascading or domino effects between disasters could be considered in future studies. In addition, visualization of non-coal underground mine risks is also a challenging task, as the large amount of information must be described in a way that is easy to understand and clear, and more helpful in guiding the specific spatial layout.

## Conclusions

1. A multi-hazard risk analysis and assessment framework for non-coal underground mines based on the inherent hazard of the system, vulnerability of the disaster-bearing body and adaptability of the disaster-bearing area is proposed. The inherent risk measurement scheme based on hazardous equipment and facilities, hazardous materials, hazardous processes, hazardous operations, hazardous places effectively solves the problem of difficult to obtain risk analysis data and standardization in the multi-hazard risk analysis of non-coal underground mines. The risk assessment framework based on inherent hazard, hazard-bearing body vulnerability and hazard-area adaptability realizes the unified measurement of risk, thus allowing comparison between different disaster types.
2. A space-oriented risk assessment model for non-coal underground mining systems exposed to a multi-hazard risk environment was constructed. Using this method, we analyzed the space-oriented multi-hazard risk of non-coal underground mines and ranked the risks of different disasters to achieve comparability of risks. The Nemerow pollution index was introduced into the multi-hazard risk aggregation of mines, which realized the regional risk aggregation and obtained the regional multi-hazard risk. This study is a space-oriented multi-hazard risk assessment method, which can be applied to both single-hazard risks and the overall regional multi-hazard risk, and can achieve the comparability of different disasters.
3. In order to improve the management level of multi-hazard risk in non-coal underground mines and reduce the probability of accidents, a "PDCA" closed-loop control model of multi-hazard risk in non-coal underground mines was established. The multi-hazard risk cooperative control system for non-coal underground mines with source identification, classification and control, process control, continuous improvement and full participation has been constructed. It provides a theoretical basis for the construction of multi-hazard risk cooperative control system for non-coal underground mines.

## Data Availability

The datasets used and analysed during the current study are available from the corresponding author on reasonable request.
